# Functional traits linked to pathogen prevalence in wild bee communities

**DOI:** 10.1038/s41598-021-87103-3

**Published:** 2021-04-06

**Authors:** Laura L. Figueroa, Sally Compton, Heather Grab, Scott H. McArt

**Affiliations:** 1 Department of Entomology, Cornell University, Ithaca, NY 14853 USA; 2 Department of Environmental Conservation, University of Massachusetts, Amherst, MA 01003 USA

**Keywords:** Community ecology, Ecological epidemiology

## Abstract

Reports of pollinator declines have prompted efforts to understand contributing factors and protect vulnerable species. While pathogens can be widespread in bee communities, less is known about factors shaping pathogen prevalence among species. Functional traits are often used to predict susceptibility to stressors, including pathogens, in other species-rich communities. Here, we evaluated the relationship between bee functional traits (body size, phenology, nesting location, sociality, and foraging choice) and prevalence of trypanosomes, neogregarines, and the microsporidian *Nosema ceranae* in wild bee communities. For the most abundant bee species in our system, *Bombus impatiens*, we also evaluated the relationship between intra-specific size variation and pathogen prevalence. A trait-based model fit the neogregarine prevalence data better than a taxa-based model, while the taxonomic model provided a better model fit for *N. ceranae* prevalence, and there was no marked difference between the models for trypanosome prevalence. We found that *Augochlorella aurata* was more likely to harbor trypanosomes than many other bee taxa. Similarly, we found that bigger bees and those with peak activity later in the season were less likely to harbor trypanosomes, though the effect of size was largely driven by *A. aurata*. We found no clear intra-specific size patterns for pathogen prevalence in *B. impatiens*. These results indicate that functional traits are not always better than taxonomic affinity in predicting pathogen prevalence, but can help to explain prevalence depending on the pathogen in species-rich bee communities.

## Introduction

The use of functional traits has been proposed as a way of increasing synthesis, generalizability, and predictability in ecology^1^, all of which are critical elements in a world characterized by accelerating global change and biodiversity loss. Functional traits are measurable properties of an organism, including morphological, physiological, and phenological characteristics, that influence performance (growth, reproduction, and survival)^2,3^. Functional traits can be effective at predicting plant community assembly, functioning, and structure^1^. Moreover, functional traits can predict how ecologically important taxa, such as bees, will respond to stressors^4,5^ and how effective their ecosystem service provisioning may be^6,7^, though not always consistently^8^. Similarly, intraspecific trait variation can affect pathogen transmission rates by influencing host susceptibility, immune response, and social behavior^9^; for example, rodents with higher body mass contribute more to tick-born encephalitis transmission than rodents with lower body mass^10^. Given the recognized role of pathogens in bee declines worldwide^11^, developing generalizable ways of predicting disease risk could help target effective conservation research.

Bee epidemiological research has historically focused on commercial bee species, especially honey bees and bumble bees, and only recently has begun to consider other bee species. Increased molecular screening efforts have found widespread pathogen prevalence in pollinator communities^12–17^, highlighting the need to understand disease dynamics at the community level. Functional traits mediate how individual bees interact with their environment, potentially influencing exposure to pathogens and likelihood of transmitting the pathogen to other bees, but to date have never been evaluated in relation to pathogen prevalence in bee communities.

Numerous bee functional traits, such as body size, phenology, nesting location, sociality, and diet could influence pathogen prevalence. Body size is correlated with foraging distance in bees^18^, and could therefore influence access to floral resources across larger spatial scales. Furthermore, bee body size not only varies substantially within species but can influence interactions with specific floral morphologies and subsequent probability of encounter with pathogens on flowers^19^. For example, the common eastern bumble bee, *Bombus impatiens*, can vary greatly both in size and in likelihood of harboring pathogens, even within the worker caste. Size can be a predictor of bee disease dynamics in this species, with bigger workers developing lower infection intensity of the trypanosomatid gut pathogen *Crithidia bombi*, independent of the dose provided^20–22^, possibly driven by a more robust immune system in larger bees^22^. As such, variation in body size among and within species could be linked to likelihood of encounter with pathogens on flowers and subsequently pathogen prevalence in bees at the community level.

Bee species differ in the time of year when they are most active as well as for how long. These phenological traits could influence pathogen transmission and prevalence. For example, bees eclose uninfected with *C. bombi*, acquiring the pathogen while foraging on contaminated flowers (social and solitary bees) or from nest mates (social bees)^19^. Bees that eclose when the incidence of disease is highest, are at greater risk of exposure, which could in turn result in greater pathogen prevalence. Whether infections accumulate or are cleared over time is not known for most bee species, nor whether patterns differ for pathogens that are exclusively transmitted horizontally, such as *C. bombi*, compared to other pathogens, such as *Nosema* spp. (microsporidia), for which sexual and vertical transmission may also be possible^23,24^. Differences in routes of transmission and persistence under varying environmental conditions could differentially drive links between phenology and prevalence of different pathogen groups.

Nesting location could also influence pathogen survival, transmission, and ultimately prevalence among bees with different nesting locations. High temperature, low moisture, and ultra-violet radiation can reduce the survival of many bee pathogens^25^. For example, *C. bombi* survives longer on flowers when shaded than when exposed to the sun^19^. These environmental conditions are known to influence choices by ground-nesting bees, and are also likely to vary between above-ground vs. below-ground nests. Some ground-nesting bees prefer warm soil surface temperatures and ample availability of solar radiation when selecting a place to nest^26,27^, which could reduce pathogen survival. However, some bees prefer moist soils for their nests^26^, or secrete water-insoluble substances into the walls of their nests to regulate moisture^28^. Moisture simultaneously increases the probability of nesting success for many ground-nesting bees while also increasing the probability for pathogen outbreaks, especially for fungal infections^29^. Thus, the nests of ground-nesting bees may facilitate the persistence and subsequent transmission of pathogens either from mother to offspring (both for social and solitary bees) or between nest mates (for social bees). While larvae of the bumble bee *B. terrestris* show no evidence of active *C. bombi* infections after inoculation, they can transmit the pathogen to brood-care worker bees, highlighting the multiple routes of pathogen transmission possible in the nest^30^. As such, ground-nesting bees may be more likely to harbor pathogens than above-ground-nesting bees.

Sociality could influence pathogen transmission and ultimately prevalence in bees, though the directionality is unclear. Group living has numerous costs and benefits related to disease susceptibility and pathogen transmission^31^. For example, social bees have evolved stronger antimicrobial defenses than their solitary counterparts^32^ and many have hygienic behaviors that reduce the probability of disease spread in the colony and increase recovery rates from infection^17,33,34^. However, social living does increase contact rates that could facilitate outbreaks within a colony; for example, social feeding (trophallaxis) and higher density of individuals are all factors that could promote disease transmission^5,25^. Furthermore, the effect of sociality on pathogen prevalence may be context dependent and be governed not only by group size but also internal organization^35^, which may vary among social bee species. In controlled experiments, having a higher initial *C. bombi* prevalence in *B. impatiens* colonies increased the final prevalence, a pattern not found for honey bees and *N. ceranae*^34^. As such, the expected relationship between sociality and pathogen prevalence in diverse bee communities is unclear.

Diet and bee foraging choices can influence pathogen transmission and prevalence. The flower species visited by bees could influence pathogen dynamics due to differences in transmission potential^19,36,37^ and/or antimicrobial properties of pollen and nectar^38,39^. In particular, sunflower pollen has been shown to markedly reduce *C. bombi* infections in bumble bees^39^. This pattern holds across numerous sunflower cultivars and at least one other species in the same plant family^40^, though patterns may be more widespread in the Asteraceae family (Figueroa, et al. unpublished data). As such, bees foraging on Asteraceae may be less likely to harbor pathogens, especially trypanosomes, than those foraging on non-Asteraceae flowers.

Here, we collected 613 bees representing 50 species collected across 11 wildflower plantings and screened them for three common bee pathogens (trypanosomes, *Nosema ceranae*, and neogregarines). We evaluated (1) taxonomic differences in pathogen prevalence, among bee families and genera, (2) temporal patterns in pathogen prevalence, (3) the relationship between bee functional traits—body size, phenology (range and peak activity), nesting location, sociality, and foraging choice—and pathogen prevalence (289 bees and 31 species), (4) intraspecific size and pathogen prevalence of the dominant species in our system *B. impatiens*, and lastly (5) the model fit based on taxonomy compared to functional traits. Infection with trypanosomes, such as *C. bombi*, can alter bumble bee foraging behavior, cognitive function^41,42^, and reproduction^43^. Similarly, the microsporidian *N. ceranae* and neogregarines such as *Apicystis bombi* can reduce adult bee survival^44–46^. Additionally, larvae inoculated with *N. ceranae* can have higher morality than sham-inoculated counterparts (mason bees)^47^ and reduced adult survival (honey bees)^48^.

## Material and methods

### Field sample collection

We collected bees foraging in 11 established wildflower plantings in central New York, USA, from June 17 to September 11, 2015 as described in Figueroa et al*.*^16^. The 10 m × 15 m plots were established in 2012 with a mix of native perennial wildflower species as well as spontaneous weedy species (in total 23 plant species and 10 plant families; Tables S1, S2). We sampled bees within the wildflower planting and adjacent areas for 1.5 ± 0.5 person-hours from 8:00 to 17:00 h at each site on sunny and low wind days, with 8 to 12 collection days per site throughout the season. Sites were sampled in alternating order so that each site was visited at different times of day throughout the season. Bees were collected while foraging on flowers and immediately placed on dry ice in the field and stored at − 80 °C until processing^16^.

### Pathogen screening

We screened for presence of *Nosema ceranae*, trypanosomes, and neogregarines in the bee guts using established Polymerase Chain Reaction (PCR) primers^16^. We surface sterilized each bee before extracting the gut, fat body, and Malpighian tubules and placing into sterile vials containing two sterilized 2.4 mm steel beads, 100 µl of sterilized 0.1 mm zirconia beads, and 800 µl of TRIsure reagent (Bioline, Boston, MA, USA). After homogenizing the samples for 30 s at 6.5 m/s using a bead mill homogenizer (Omni International, Kennesaw, GA, USA), we transferred the solution to a new sterile vial, taking care to not pipette beads or large tissue fragments. We extracted DNA following the manufacture’s protocol. Each extraction batch included a negative control. The PCR primers are provided in Table S3. While the primers for trypanosomes and neogregarines were developed to be taxonomically inclusive, upon Sanger sequencing a subset of the samples from numerous bee species we found that 66% of the trypanosome positive samples had very high sequence similarity with *C. bombi* (others present were *C. mellificae*, *Herpetomonas pessoai*, and Trypanostomatidae sp.) and that 100% of the neogregarine positive samples had very high sequence similarity with *A. bombi*^16^.

### Bee identification

To reduce risk of DNA degradation and cross-contamination, we identified the bee species post-dissection. As such, not all specimens retained the characters necessary for identification. Bees were predominantly identified using reference materials located in the Cornell University Insect Collection and published keys^49–54^ (additional information in Figueroa et al*.* 2020). Bees in the genus *Lasioglossum* are difficult to identify morphologically and were thus identified using barcoding methods. Specifically, we amplified and sequenced a 900 bp region of the protein-coding gene elongation factor 1-alpha^16^. Ultimately, we were able to identify 86% of all bees to species, 13% to genus only, and unable to identify 1% of the bees. Unidentified bees were removed from the analyses (*n* = 8). To be conservative, we conducted analyses only for bees that amplified positive control (bee DNA) or pathogen DNA (separate because multiplex primers developed to favor pathogen amplification).

### Functional trait assessment

We selected six functional traits that we hypothesized could be important for determining pathogen prevalence: body size (continuous; empirically determined), phenology (continuous; peak and range), nesting location (above-/below- ground), sociality (social/solitary), and foraging choice (Asteraceae/non-Asteraceae). These traits are frequently used in the bee literature^8,55,56^ and we were able to obtain information for many of the species in our study (Table S4). We determined body size by measuring the intertegular distance (ITD), defined as the distance between the bees’ wing bases (Cane, 1987; Greenleaf et al., 2007) using an Olympus SZX10 microscope and CellSens Standard software (Olympus Corporation of the Americas, Scientific Solutions Group, Waltham, Massachusetts, USA). Due to risk of cross-contamination and DNA-degradation, we measured ITD post-dissection. As such, some specimens were not in suitable condition for adequately measuring ITD (76 individuals). To include the specimens that were not directly measured in community-wide analyses (but not intraspecific analyses), we used the average ITD of other bees of the same species and sex at the same site (reduced number of individuals without measurement to 35). Bee phenology was obtained from a publicly available database of Northeastern US bees^56^. Specifically, we evaluated mean Julian day of adult activity as well as phenological range of activity (90th quantile—10th quantile in Julian date of adult activity), hereafter referred to as peak seasonal activity and seasonal activity range.

To avoid using nesting-trait categories that would have been represented by few species, we classified stem- and wood-nesting as “above-ground-nesting”, compared to ground-nesting bees as described in Williams et al. 2010. We also consolidated sociality according to Forrest et al. 2015, in which social bees include communal and facultative social species, as compared to obligate solitary species; we collected a single brood parasite (*Coelioxys rufitarsis*), and so we did not include brood parasites as a separate category. We evaluated the plant family on which the bee was collected, and characterized it as either Asteraceae or non-Asteraceae, hereafter referred to as foraging choice. We made this selection both because there were low number of representatives for many plant families and because of evidence that many Asteraceae have antimicrobial properties against bee pathogens (Figueroa et al. unpublished data). The full list of bee species and traits is in Table S4. Honey bees were only collected in the latter half of the sampling period^16^; however, given that there were no temporal trends in pathogen prevalence (see “Results”), we feel confident that this did not influence our results.

### Statistical analyses

Statistical analyses were conducted in RStudio (R Core group, version 3.4.0) using the lme4 and epiR packages^57,58^. We computed the true pathogen prevalence across all sites for each of the three pathogen groups (trypanosomes, *N. ceranae*, and neogregarines; *n* = 613) using the *epi.prev* function of the epiR package, which accounts for differences in abundance. The test sensitivity was set to 95% using the Blaker method for two-sided confidence intervals^58^. This method was also used to evaluate pathogen prevalence for the most abundant species, *Bombus impatiens* (*n* = 106). We evaluated temporal patterns of pathogen prevalence using generalized linear mixed effects models (GLMM), which included pathogen status (proportion of pathogen positive bees out of the bees screened at each site) as a binomial response, month as the numeric explanatory variable, and site as a random effect. We conducted a likelihood ratio test to determine the significance of coefficients by comparing against a null model that only included the random effect (site). The unit of replication was the number of unique site-month combinations in which we sampled and screened bees (*n* = 40). All pathogen prevalence analyses included a binomial distribution and logit-link as transformation.

Before evaluating the relationship with functional traits, we evaluated whether there were taxonomic signals in pathogen prevalence in the bee communities. We constructed GLMMs that included pathogen presence/absence in individual bees as the binary response (conducted separately for trypanosomes, *N. ceranae*, and neogregarines), with bee family as the explanatory variable and bee species and site as random effects. The models included sex as a covariate. To determine significance, we conducted a likelihood ratio test that compared the model including family as the predictor to one that only included the random effects (*n* = 466 bees). We similarly evaluated differences among bee genera. We used family and genus as the predictors instead of species because some genera and species only included very few representative individuals (sometimes only one; Table S4). When the model was significant, a post-hoc test was used to determine significant differences among families or genera (Tukey’s HSD).

We evaluated the relationship between bee pathogen prevalence and bee functional traits using a GLMM that included pathogen presence/absence in individual bees as the binomial response predicted by each of the six traits (size, peak seasonal activity, seasonal activity range, nesting location, sociality, and foraging choice), including species and site as the random effects. Given that female and male bees can vary in size^59^ and can both become infected^60–63^, models included sex as a covariate. While we successfully screened 613 identified bees, we analyzed all traits simultaneously in a single GLMM and hence only used species without missing trait or sex data (*n* = 289 individuals). We conducted this analysis separately for each pathogen group (trypanosomes, *N. ceranae*, and neogregarines). Including sociality raised the VIF above 2; once this factor was removed from the model, the VIF was less than two for all remaining factors and were therefore analyzed simultaneously^64^. Based on the taxonomic analysis, we found that *Augochlorella aurata* was significantly more likely to have trypanosomes than other genera (this species was the only representative of the genus in our system; see “Results”). To determine whether patterns were driven by this species, we excluded *A. aurata* from the datasheet and re-analyzed significant models. To further evaluate whether season could be driving size patterns, we evaluated differences in bee size over the sampling period by constructing a Linear Mixed Model that included bee size (ITD) as the response predicted by month, including species and site as random effects. Bee size was log-transformed to meet assumptions of normality.

We evaluated whether intraspecific body size predicted presence of each of the pathogens in the dominant species (*Bombus impatiens*, *n* = 106 workers). This was the only species for which we had sufficient numbers to evaluate intra-specific trait variation (*i.e.* ≥ 50 individuals^1^). To address this question, we conducted a GLMM that included pathogen presence as the binary response, predicted by individual bee size, and site as the random effect; this was done separately for each of the three pathogen groups. Significance was determined using likelihood ratio test that compared the model including bee size as a predictor to a null model that only included the random effect (site). The analysis was conducted only on worker bees, as no queens or drones were collected. The intra-specific size variation followed a normal distribution, with the exception of one very small worker (Fig. S1). Excluding the single outlier had a large effect on the results (the only significant pattern was for *N. ceranae*, which changed from *P* = 0.042 to *P* = 0.275 when the outlier was removed). Although we have no reason to suspect this data point was incorrectly measured, here we choose not to highlight this result since it is does not appear to be a general pattern (Fig. S2). As such, in the main text we present the results with the single outlier removed from the analyses.

Lastly, we compared the fit of models that included functional traits as predictors compared to taxonomic groupings (genus) using Akaike Information Criterion (AIC). To do this, we compared GLMMs that included pathogen presence as the response (trypanosome, *N. ceranae*, or neogregarine) predicted by the six functional traits or genus; both models included species and site as random effects and were evaluated on the dataset used in the functional trait analyses (*n* = 289). Both models included sex as a covariate. Differences in AIC were used to rank the fit of models to the data, and ∆AIC ≥ 2 were taken to indicate substantial differences between models^65^.

## Results

**Figure 1:**
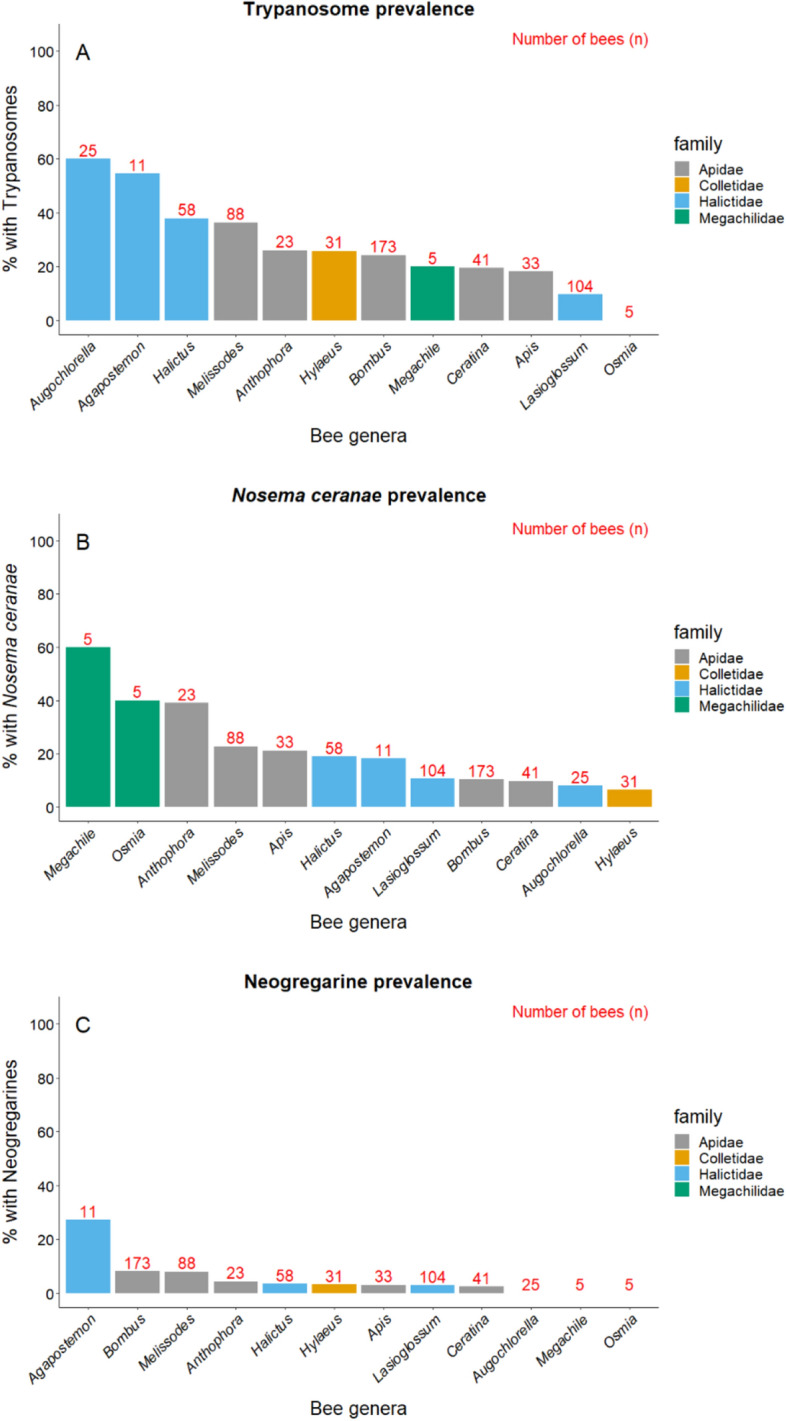
Prevalence of (**A**) trypanosomes, (**B**) *Nosema ceranae*, and (**C**) neogregarines among bee genera. The genera are ordered by decreasing prevalence within families. Differences were significant (*P* < 0.05) for trypanosomes and *N. ceranae* (post-hoc comparisons in Table S4). Genera with fewer than 5 individuals screened are excluded from figure.

**Figure 2:**
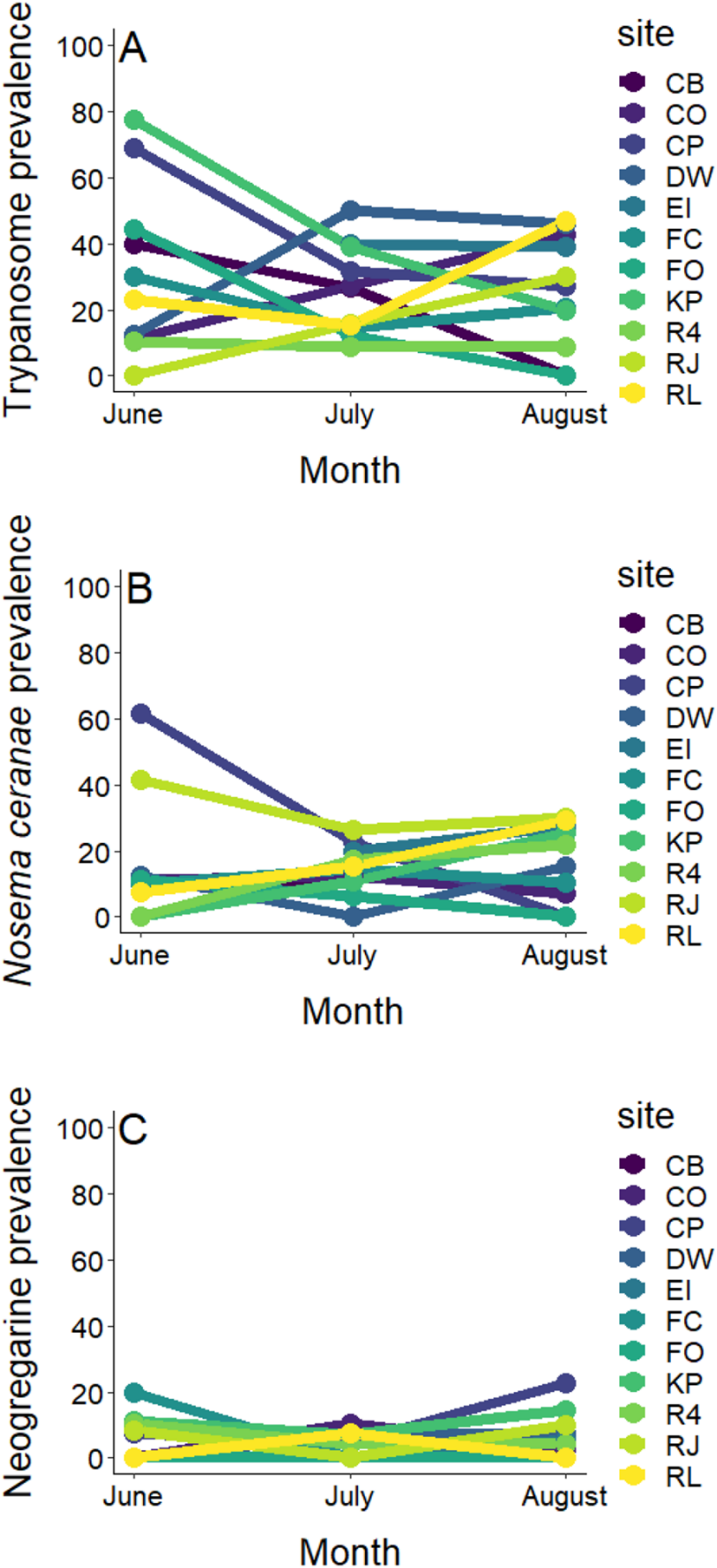
Temporal patterns of pathogen prevalence in the bee communities. Sampling dates where fewer than five bees were collected are not shown, nor are September sampling dates as only three sites had more than 5 bees collected then. There were no overall significant temporal patterns in prevalence for any of the three pathogen groups screened (see “Results”).

Pathogen prevalence in the bees ranged from 23% for trypanosomes (95% Confidence Interval (CI), 20–27%), to 11% for *Nosema ceranae* (95% CI 8–15%), and 1% for neogregarines (95% CI 0–3%). Pathogen prevalence varied among bee genera for trypanosomes (χ^2^_19_ = 30.80, *P* = 0.042; Fig. 1A) and *N. ceranae* (χ^2^_19_ = 40.08, *P* = 0.003; Fig. 1B), though not neogregarines (χ^2^_19_ = 22.11, *P* = 0.28; Fig. 1C). *Post-hoc* tests indicate that bees in the genus *Augochlorella* (of which there was only one species in our system *A. aurata*) were more likely to harbor trypanosomes than those in the genera *Lasioglossum* (*z* = 4.16, *P* = 0.005), *Bombus* (*z* = 3.85, *P* = 0.017), and *Apis* (*z* = 3.61, *P* = 0.040; Fig. 1). Other comparisons between genera were not significant for trypanosomes, nor were comparisons for *N. ceranae* prevalence (*P* > 0.05; Table S5). There were no differences among bee families for any of the three pathogens screened (χ^2^_4_ = 2.83, *P* = 0.59, χ^2^_4_ = 6.87, *P* = 0.14, χ^2^_4_ = 5.92, *P* = 0.21 for trypanosome, *N. ceranae*, and neogregarine prevalence, respectively). Month did not explain pathogen prevalence in our bee communities (June–September) (χ^2^_1_ = 0.04, *P* = 0.84, χ^2^_1_ = 0.00, *P* = 0.96, χ^2^_1_ = 0.58, *P* = 0.45 for trypanosome, *N. ceranae*, and neogregarine prevalence, respectively), indicating no clear temporal pattern of pathogen prevalence across sites, despite marked differences across time within sites (Fig. 2).

**Figure 3:**
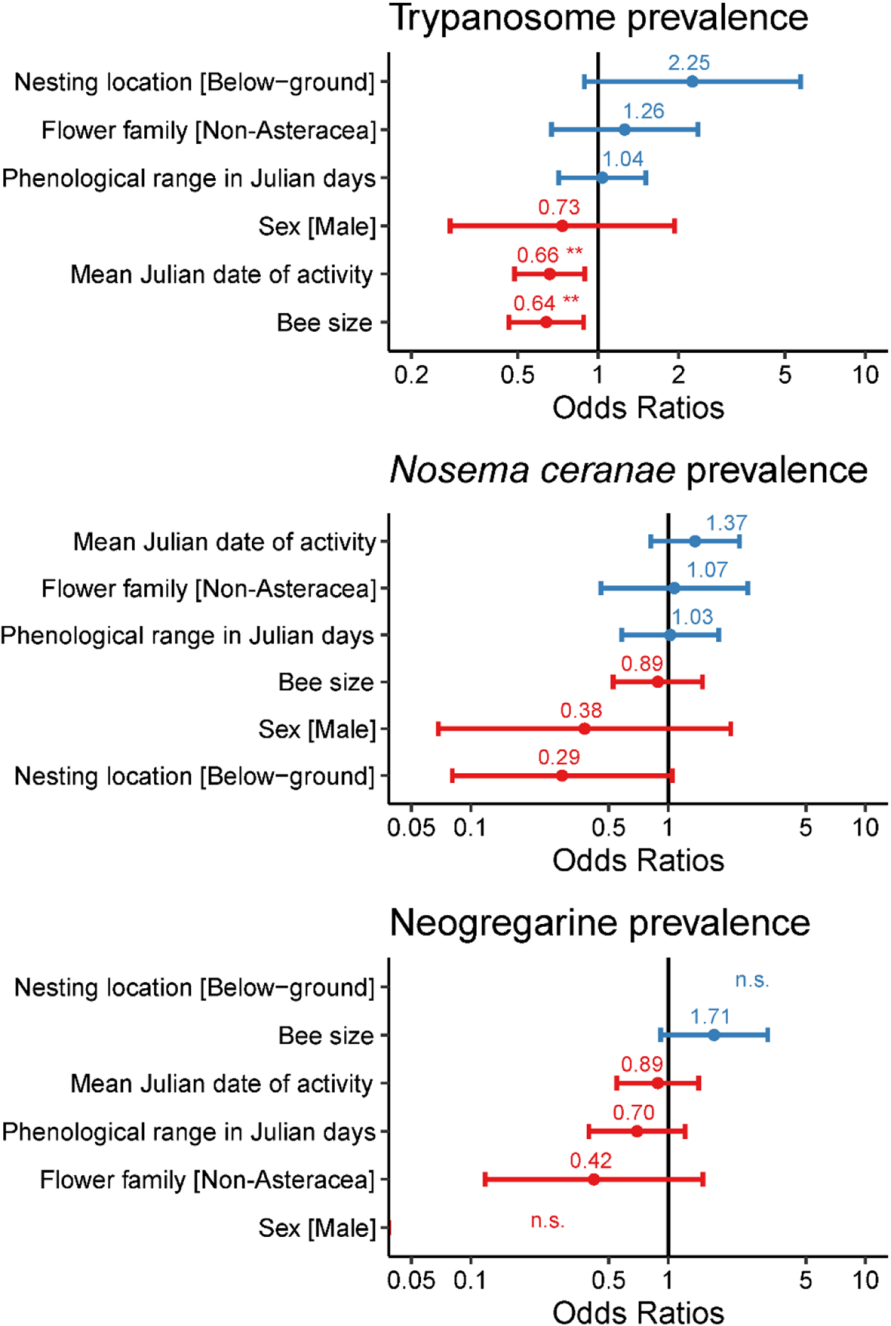
Odds Ratios from binomial models evaluating pathogen prevalence in bee communities by functional traits. Factors sorted from highest to lowest value. Blue indicates a positive odds ratio and red indicates a negative odds ratio. ** *P* < 0.01. For neogregarines, sex and nesting locations had odds ratios and errors orders of magnitude bigger than other factors, so for visualization n.s. included to indicate they were not significant.

**Figure 4:**
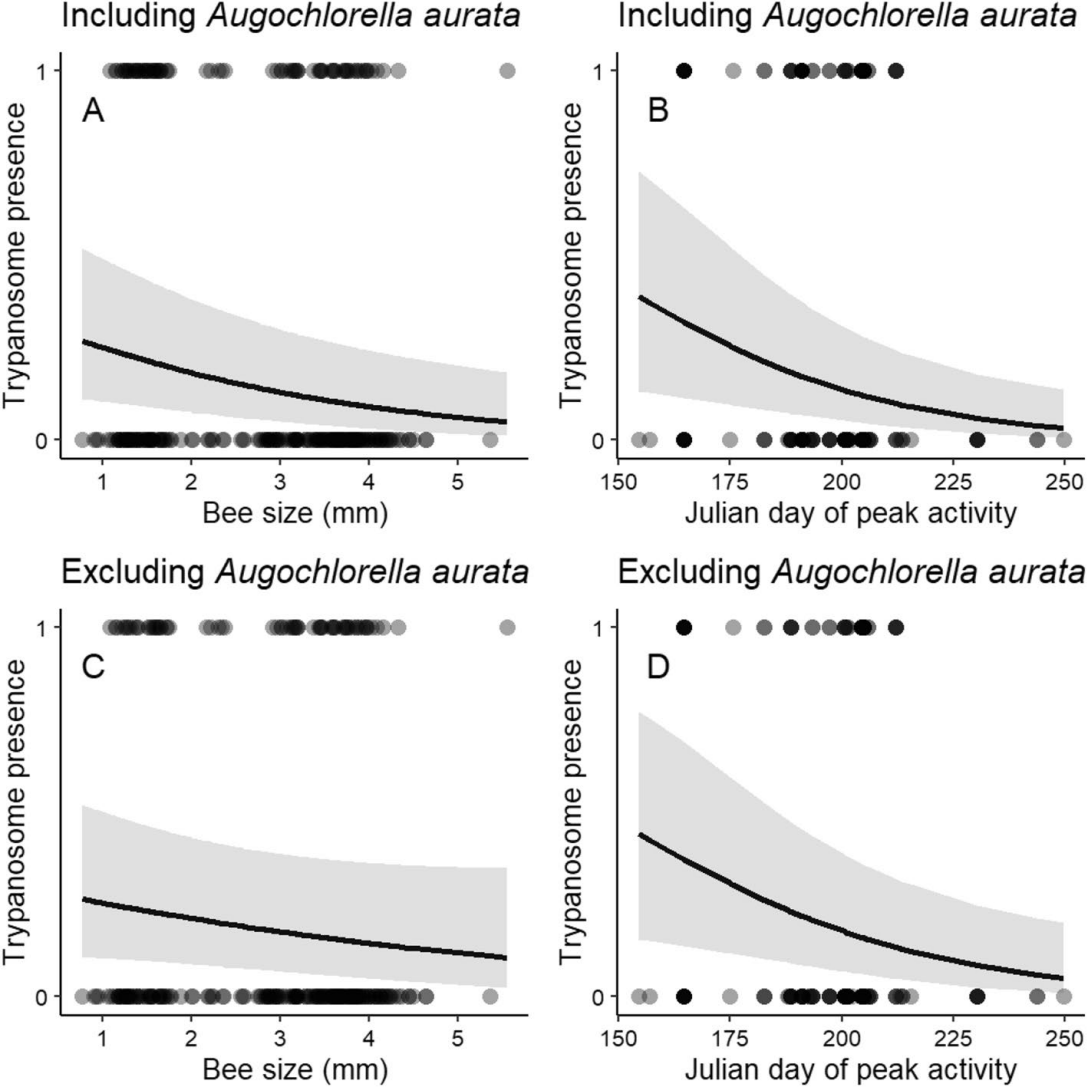
Relationship between bee size and trypanosome prevalence in the overall community (**A**) when *A. aurata* included and (**B**) when *A. aurata* excluded. Relationship between peak seasonal activity (in Julian days) and trypanosome prevalence (**C**) when *A. aurata* included and (**D**) when *A. aurata* excluded. Shaded confidence interval corresponds to standard error. The regression lines are based on the model output (Table S6). Data are presented as proportions for ease of visualization in Fig. S3.

Functional traits were linked to trypanosome prevalence, though not to *N. ceranae* and neogregarine prevalence (Fig. 3). Bigger bees and those that were active later in the season were less likely to harbor trypanosomes than smaller counterparts and those active earlier in the season (*z* = − 2.72, *P* = 0.006 and *z* = − 2.72, *P* = 0.007, for bee size and peak seasonal activity, respectively; Figs. 4A,B, S2). We excluded *A. aurata* from the analyses to evaluate whether its disproportionately high trypanosome prevalence was driving the patterns, as a comparatively smaller and earlier-active species (Table S4). We found that *A. aurata* was largely driving the effects of size (*z* = − 1.46, *P* = 0.144 when *A. aurata* removed; Fig. 4C), though the peak seasonal activity patterns remained largely unchanged (*z* = − 2.49, *P* = 0.013; Fig. 4D). We found no seasonal differences in bee size as delineated by month (χ^2^_1_ = 1.69,* P* = 0.19). Phenological range, nesting location, sex and foraging choice (whether or not collected on Asteraceae) did not explain trypanosome prevalence in the bees (Table S6), nor was any trait linked to *N. ceranae* or neogregarine prevalence (Table S6).

We evaluated the role of intra-specific size variation in relation to pathogen prevalence in *Bombus impatiens,* which ranged from 19% for trypanosomes (95% CI 10–28%), to 11% for *N. ceranae* (95% CI 5–20%), and 5% for neogregarines (95% CI 0–13%). Pathogen prevalence was not linked to intraspecific size in *B. impatiens* workers (χ^2^_1_ = 0.10,* P* = 0.75, χ^2^_1_ = 1.19,* P* = 0.27, and χ^2^_1_ = 0.57, *P* = 0.45, for trypanosomes, *N. ceranae* and neogregarines respectively).

The comparison of model fit between functional traits compared to taxonomic-based models yielded inconsistent results. Models that included functional traits as explanatory variables had a better model fit compared to the taxonomic-based (genus) model for neogregarine prevalence (∆AIC = − 12.36), worse fit for *N. ceranae* (∆AIC = 12.13), and was not different for trypanosome prevalence (∆AIC = 1.06).

## Discussion

Taxonomic affinity and functional traits have both been successfully used to predict susceptibility to pathogen prevalence in species-rich communities, though these frameworks have not yet been applied to understand host–pathogen dynamics in naturally occurring pollinator communities. Here we found that pathogen prevalence varied among bee genera for trypanosomes and *Nosema ceranae* in the bee communities surveyed, though interestingly no differences were found among bee families for any of the pathogens screened. There were no clear temporal patterns in pathogen prevalence across the bee communities. Conversely, we found that bigger bees and those that have a later peak season of activity were less likely to harbor trypanosomes, such as *Crithidia bombi*. The size pattern was driven by *Augochlorella aurata*, a species with disproportionately high trypanosome prevalence. This species, however, was not the driver of the phenological pattern, which remained after this species was excluded from analyses. Other functional traits were not linked to trypanosomes, and nor were any linked to *N. ceranae* or neogregarine prevalence. For the dominant species in the communities, *B. impatiens*, there was no clear relationship between intraspecific size and pathogen prevalence for any of the three pathogen groups screened. Overall, our work highlights that specific functional traits can explain pathogen prevalence patterns in diverse pollinator communities and pinpoints future areas for research in bee epidemiology.

We found taxonomic signals in pathogen prevalence, finding that trypanosome and *N. ceranae* prevalence varied by bee genera (Fig. 1). Bees in the genus *Augochlorella*, which were all *A. aurata* in our bee communities, were more likely to harbor trypanosomes than those in *Lasioglossum*, *Bombus*, and *Apis*. This finding supports a previous study that found high prevalence of the trypanosome *C. bombi* in *Augochlorella*^17^, suggesting additional experimental work should be conducted to understand the importance of this genus as a host, including assessing health consequences of infection and transmission potential on flowers. There were no significant differences in neogregarine prevalence among genera, nor did comparisons between any two genera yield significant patterns for *N. ceranae* prevalence. Similarly, we found no differences in pathogen prevalence among bee families, indicating that there may be key players at the species level that vary markedly from community level patterns. It is important to note that pathogen prevalence was determined using molecular methods, and as such, we cannot distinguish between innocuous pathogen DNA and active infections, nor whether patterns are due to greater resistance or less exposure to the pathogens. This work highlights the need to experimentally assess the impacts of these pathogens beyond honey bees and bumble bees^62,63^, and determine which bee species, such as *A. aurata*, play key roles in pathogen transmission at the community level^16,66^.

Trypanosome prevalence was linked to functional traits in the bee communities. Specifically, bigger bees and those with a later peak season of activity were less likely to harbor trypanosomes (Fig. 4A,B). Given the disproportionately high trypanosome prevalence in *A. aurata*, a species that is relatively small and has a peak season of activity earlier in the summer, we wanted to evaluate whether the patterns remained when this species was excluded from the analyses. We found that there was no longer an effect of bee size, indicating that *A. aurata* was largely responsible for the effect of this trait (Fig. 4C). Conversely, the pattern of bees with a later peak seasonal activity remained (Fig. 4D). Interestingly, this occurred despite no clear temporal patterns of pathogen prevalence over time across sites (Fig. 2), nor significant differences in bee size over time. There are often fewer bee species later in the season, as can be seen not only in our data but also more generally in the bee species community of the Northeastern United States (Fig. S4), and bee species likely differ in deposition rates on flowers^66^. Pathogen prevalence is likely influenced by the number of competent hosts with broadly overlapping phenologies, as transmission on flowers depends on short time periods between an infected individual depositing pathogens on flowers and susceptible incomers foraging on the contaminated flowers^19^. If there are fewer competent hosts and/or fewer interactions with the same flowers later in the season, the probability of transmission may be lessened, subsequently reducing prevalence. Further evaluations into the role of phenology, visitation frequency, and overlapping use of floral resources in bee disease dynamics is clearly warranted^16,17^.

Intra-specific variability plays a key role in driving species coexistence, ecosystem functioning, and response to global change, which can be comparable to or even greater than inter-species differences^1,67^. Individual body size was not linked to increased likelihood of harboring any of the three screened pathogens in the dominant bee species in our study, *Bombus impatiens*. Other studies have found that bigger individuals of this species are less likely to harbor the trypanosomatid pathogen *C. bombi*, possibly due to a more robust immune system^20–22^. However, many studies evaluating the relationship between bee size and pathogens have been conducted in the laboratory where bees have access to pollen and sucrose ad libitum. In the field, bees experience numerous interacting stressors (*e.g.* decreased floral diversity, increased pesticide exposure, and competition with honey bees^68^), often being smaller in more simplified agricultural landscapes^69–72^. As such, we do not know if small individuals that were infected succumbed to the negative health consequence of the pathogens (and therefore were not out foraging for us to collect and screen), while larger individuals could tolerate the infections and thus bias the patterns. Additionally, smaller bumble bees often remain in the hive as brood-care workers while larger bees forage^73^, with the potential to further bias the sampling. A recent observational study similarly found no relationship between bee size and pathogen prevalence in wild bumble bee workers^74^. As such, determining whether landscape scale filtering of bees could increase susceptibility to infections is a necessary future research direction, and highlights the importance of experimental manipulations in the field with co-occurring stressors^75^.

Not all functional traits were associated with differences in pathogen prevalence in the bee communities, nor were there sex differences (Fig. 3). Namely, range of activity period, nesting location, foraging choice (whether collected or not on Asteraceae) were not linked to pathogen prevalence in the bees (sociality was highly collinear with the other functional traits and thus not included in the final model). The comparison between the model fit of the functional trait model and the taxonomy (genus) model differed markedly by pathogen group. Interestingly, while two traits were significantly linked to trypanosome prevalence, there was no difference in model fit between the functional traits and the taxonomy models for this pathogen group. Conversely, the functional trait model provided a significantly better model fit for neogregarines and a significantly worse model fit for *N. ceranae*. In a previous study, trait-based models evaluating trypanosome (*C. bombi*) transmission to *B. impatiens* at flowers had lower AIC values than models based on plant taxonomy^37^. Diverse pollinator communities are often characterized by many rare species^76^, and thus employing functional traits may provide a better model fit by requiring fewer parameters, though as our study shows, not always. Given the recognized role of pathogens in bee declines worldwide^11^ and their widespread prevalence in surveyed plant-pollinator communities^12,13,16^, developing generalizable ways of predicting disease could guide more effective conservation efforts. The negative relationship between bee size (foraging distance) and peak seasonal activity (lower species richness) indicate that experimentally testing whether greater number of flowers could reduce the overlapping use of floral resources and subsequently reduce rates of pathogen transmission may be a fruitful future direction. Furthermore, collecting phenological data through long-term monitoring efforts of bee communities is very important as patterns may shift with climate change^77^, which may be shaped by other functional traits of the bees^78^, with implications for access to resources, pathogen transmission, and ultimately pollinator health. Our work illustrates that there are taxonomic and functional trait signals in the prevalence of some pathogen groups but not others in diverse pollinator communities.

## Supplementary Information

Supplementary Information.
