# Supplementary material for: Functional traits linked to pathogen prevalence in wild bee communities
Source: Sci Rep. 2021 Apr 6;11:7529. doi: 10.1038/s41598-021-87103-3 (PMC8024325; doi:10.1038/s41598-021-87103-3)
Supplement: Supplementary file 1 — Supplementary Information. [file 41598_2021_87103_MOESM1_ESM.docx]

**Functional Traits Linked to Pathogen Prevalence in Wild Bee Communities**

**Supplementary Material**

**Authors:** Laura L. Figueroa^1,2^* [llf44@cornell.edu](mailto:llf44@cornell.edu)

Sally Compton^1^  [sallymcompton@gmail.com](mailto:sallymcompton@gmail.com)

Heather Grab^1^ [hlc66@cornell.edu](mailto:hlc66@cornell.edu)

Scott H. McArt^1^ [shm33@cornell.edu](mailto:shm33@cornell.edu)

^1^Department of Entomology, Cornell University, Ithaca, NY 14853, USA

^2^Department of Environmental Conservation, University of Massachusetts, MA, 01003, USA

*Corresponding author: Laura L. Figueroa

**Figure S1.** Histogram showing distribution of bee size in *B. impatiens* workers. One clear outlier was removed from the *B. impatiens* intraspecific statistical analyses (furthest to the left).


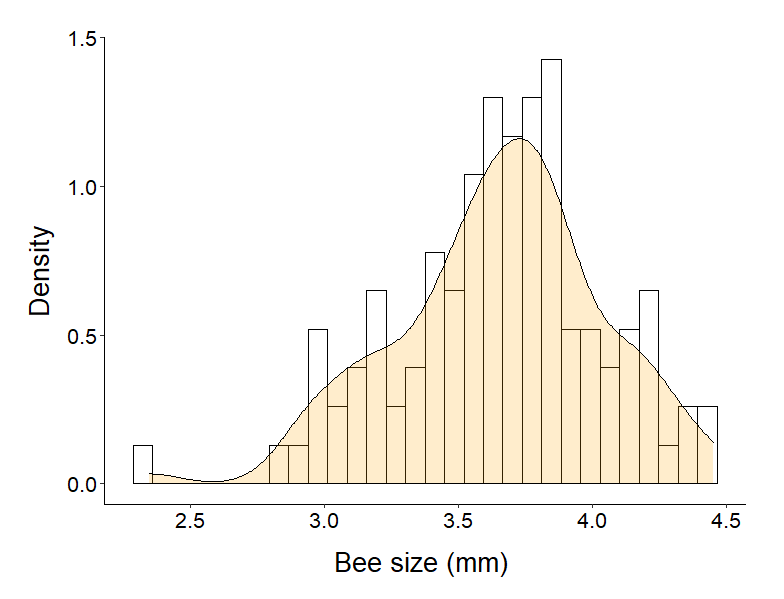


**Figure S2.** Relationship between individual *Bombus impatiens* worker size and likelihood of *Nosema ceranae* presence A) for all bees sampled and B) excluding theoutlier (Figure S1). **
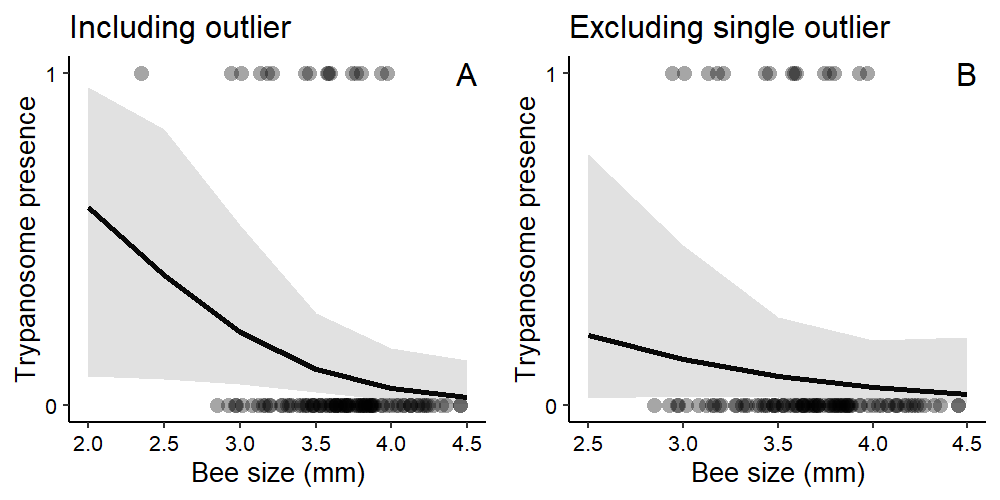
**

**Figure S3.** Relationship between bee size and trypanosome prevalence (mean ±SE) in the overall community (A) when *A. aurata* included and (B) when *A. aurata* excluded. Relationship between peak seasonal activity (in Julian days) and trypanosome prevalence (mean ±SE) in the overall community (A) when *A. aurata* included and (B) when *A. aurata* excluded. Data analyzed as continuous and plotted as such in Figure 4, presented here as proportion for ease of visualization.**
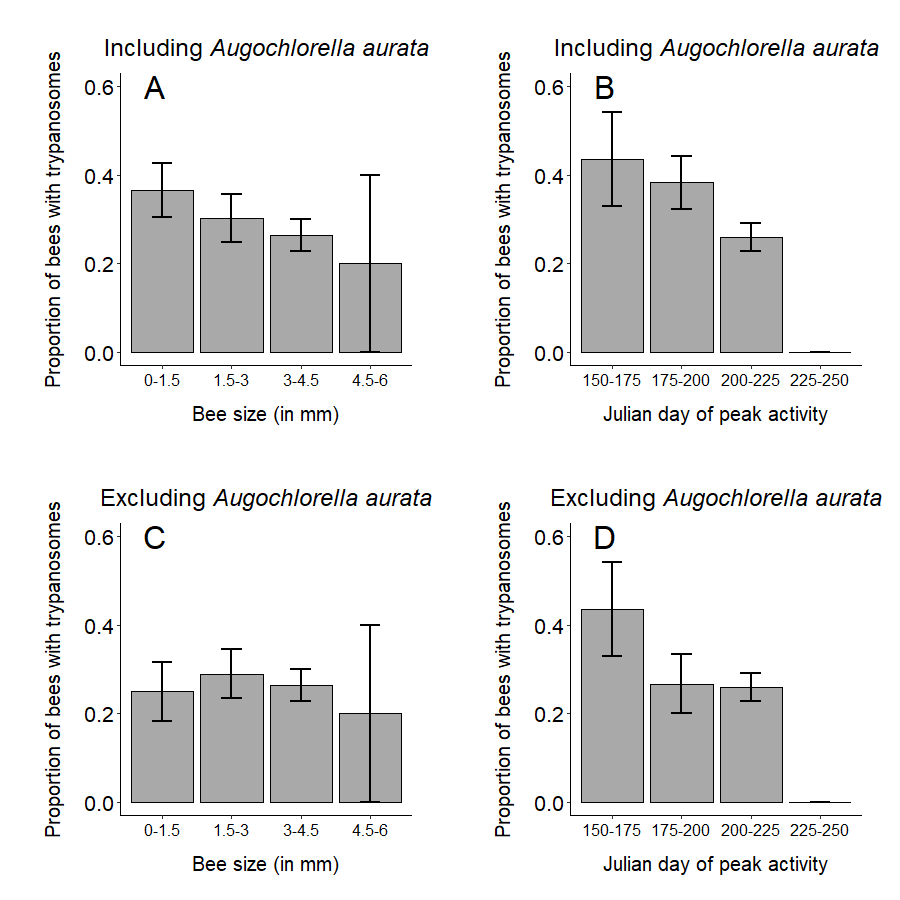
**

**Figure S4**. Histogram showing distribution of peak seasonal activity (based on Julian day) for the samples included in the functional trait analyses (A) and for the species in the NE of the US (overlapping region with the present study)^1^.


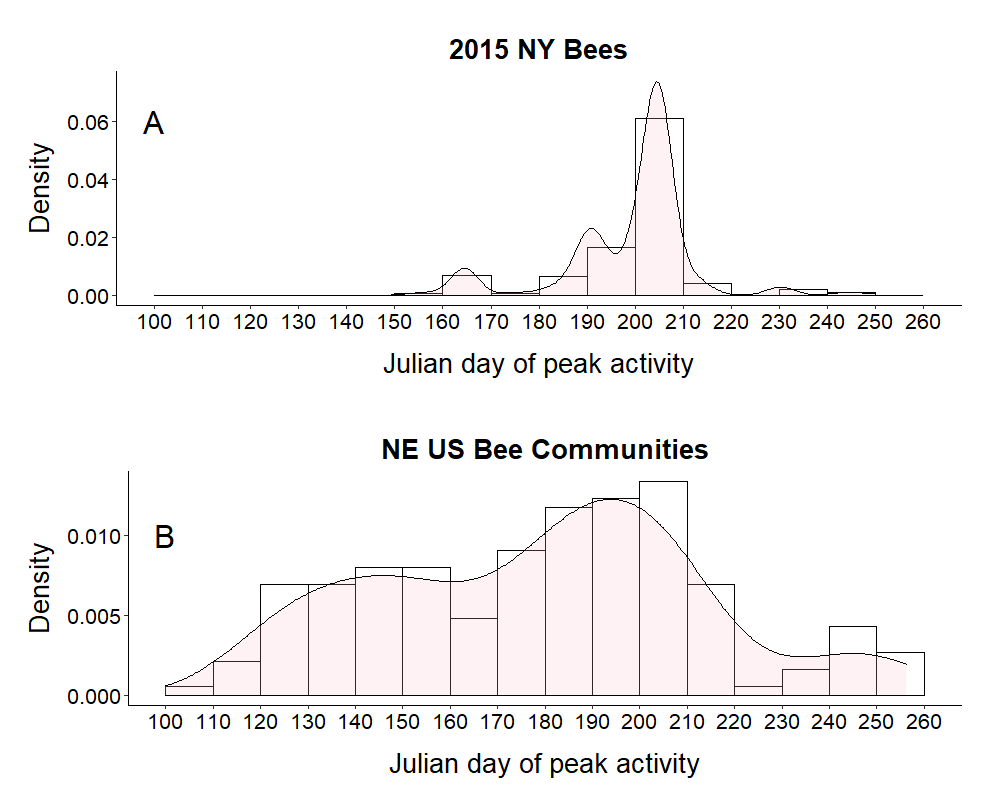


**Table S1:** Flower species present in wildflower planting and adjacent area where bees were collected.

| **In planting** | **Adjacent** |
| --- | --- |
| *Agastache nepetoides* | *Convolvulus arvensis* |
| *Cirsium arvense* | *Daucus carota* |
| *Coreopsis lanceolata* | *Hypericum perforatum* |
| *Daucus carota* | *Leucanthemum vulgare* |
| *Erigeron annuus* | *Lotus corniculatus* |
| *Lobelia siphilitica* | *Malva moschata* |
| *Medicago sativa* | *Potentilla fruticosa* |
| *Penstemon digitalis* | *Plantago lanceolata* |
| *Silphium perfoliatum* | *Rubus fruticosus* |
| *Solidago juncea* | *Symphyotrichum novi-belgii* |
| *Trifolium pratens* |  |
| *Trifolium repens* |  |
| *Veronicastrum virginicum* |  |

**Table S2:** Plant families visited by each bee species across the 11 sampled sites.

| Bee species | Plant Family | *n* |
| --- | --- | --- |
| *Agapostemon virescens* | Asteraceae | 4 |
|  | Plantaginaceae | 7 |
| *Andrena simplex* | Asteraceae | 1 |
| *Andrena spp.* | Asteraceae | 1 |
| *Andrena wilkella* | Fabaceae | 2 |
| *Anthophora terminalis* | Plantaginaceae | 22 |
|  | NA | 1 |
| *Apis mellifera* | Asteraceae | 33 |
| *Augochlora pura* | Plantaginaceae | 2 |
| *Augochlorella aurata* | Asteraceae | 9 |
|  | Convolvulaceae | 6 |
|  | Fabaceae | 2 |
|  | Plantaginaceae | 8 |
| *Bombus bimaculatus* | Asteraceae | 3 |
|  | Fabaceae | 4 |
|  | Plantaginaceae | 16 |
| *Bombus fervidus* | Asteraceae | 1 |
|  | Fabaceae | 1 |
|  | Plantaginaceae | 1 |
| *Bombus griseocollis* | Asteraceae | 4 |
|  | Plantaginaceae | 5 |
| *Bombus impatiens* | Apiaceae | 2 |
|  | Asteraceae | 99 |
|  | Campanulaceae | 3 |
|  | Fabaceae | 1 |
|  | Lamiaceae | 5 |
|  | Plantaginaceae | 12 |
| *Bombus perplexus* | Asteraceae | 1 |
| *Bombus ternarius* | Asteraceae | 3 |
|  | Plantaginaceae | 2 |
| *Bombus vagans* | Asteraceae | 3 |
|  | Fabaceae | 1 |
|  | Plantaginaceae | 6 |
| *Calliopsis andreniformis* | Asteraceae | 1 |
| *Calliopsis nebraskensis* | Asteraceae | 1 |
| *Ceratina calcarata* | Asteraceae | 5 |
|  | Campanulaceae | 3 |
|  | Malvaceae | 1 |
|  | Plantaginaceae | 3 |
|  | Rosaceae | 1 |
|  | *NA* | 2 |
| *Ceratina dupla* | Plantaginaceae | 1 |
|  | Rosaceae | 1 |
| *Ceratina mikmaqi* | Asteraceae | 8 |
|  | Fabaceae | 1 |
|  | Hypericaceae | 1 |
|  | Plantaginaceae | 8 |
| *Ceratina spp.* | Asteraceae | 2 |
|  | Campanulaceae | 1 |
|  | Plantaginaceae | 3 |
| *Coelioxys rufitarsis* | Asteraceae | 1 |
| *Colletes kincaidii* | Asteraceae | 1 |
| *Halictus confusus* | Asteraceae | 13 |
|  | Convolvulaceae | 1 |
|  | Plantaginaceae | 2 |
|  | Rosaceae | 1 |
| *Halictus ligatus* | Asteraceae | 32 |
|  | Convolvulaceae | 8 |
| *Halictus rubicundis* | Fabaceae | 1 |
| *Heriades carinata* | Asteraceae | 1 |
| *Hoplitis producta* | Plantaginaceae | 1 |
| *Hoplitis spp.* | Asteraceae | 1 |
| *Hylaeus affinis* | Asteraceae | 6 |
|  | Convolvulaceae | 2 |
|  | Plantaginaceae | 3 |
| *Hylaeus annulatus* | Asteraceae | 1 |
|  | Malvaceae | 1 |
|  | Plantaginaceae | 1 |
| *Hylaeus modestus* | Asteraceae | 1 |
|  | Campanulaceae | 9 |
|  | Plantaginaceae | 4 |
| *Hylaeus spp.* | Asteraceae | 3 |
| *Lasioglossum coriaceum* | Plantaginaceae | 3 |
| *Lasioglossum hitchensi* | Plantaginaceae | 1 |
| *Lasioglossum imitatum* | Asteraceae | 2 |
| *Lasioglossum inconditum* | *NA* | 1 |
| *Lasioglossum lineatulum* | Asteraceae | 1 |
|  | Plantaginaceae | 2 |
| *Lasioglossum perpunctatum* | Asteraceae | 1 |
| *Lasioglossum pilosum* | Asteraceae | 4 |
|  | Plantaginaceae | 7 |
| *Lasioglossum spp.* | Asteraceae | 10 |
|  | Convolvulaceae | 1 |
|  | Hypericaceae | 4 |
|  | Plantaginaceae | 10 |
| *Lasioglossum versatum* | Plantaginaceae | 3 |
| *Lasioglossum viridatum* | Asteraceae | 7 |
|  | Convolvulaceae | 4 |
|  | Fabaceae | 1 |
|  | Plantaginaceae | 31 |
|  | Rosaceae | 2 |
|  | *NA* | 1 |
| *Lasioglossum weemsi* | Campanulaceae | 1 |
| *Lasioglossum zephyrum* | Asteraceae | 3 |
|  | Hypericaceae | 3 |
|  | Plantaginaceae | 1 |
| *Megachile spp.* | Asteraceae | 1 |
|  | Plantaginaceae | 4 |
| *Melissodes agilis* | Asteraceae | 5 |
| *Melissodes bimaculata* | Asteraceae | 4 |
| *Melissodes desponsa* | Asteraceae | 6 |
| *Melissodes druriella* | Asteraceae | 3 |
| *Melissodes spp.* | Asteraceae | 37 |
|  | *NA* | 1 |
| *Melissodes subillata* | Asteraceae | 3 |
| *Melissodes trinodis* | Asteraceae | 29 |
| *Osmia atriventris* | Plantaginaceae | 1 |
| *Osmia bucephala* | Plantaginaceae | 4 |
| *Peponapis pruinosa* | Asteraceae | 1 |
| *Xylocopa virginica* | Asteraceae | 2 |

**Table S3. Molecular conditions used for pathogen screening (PCR).**

| Primers and source | Thermal Cycling | | | Amplicon size (bp) |
| --- | --- | --- | --- | --- |
|  | Denaturing Min \| Temp | Replication Sec \| Temp | Elongation Min \| Temp |  |
|  |  |  |  |  |
| ***Apidae^A^ (host) and Trypanosomes^T^ ^2^*** | 2 \| 94 | 35x 30 \| 94 30 \| 61 45 \| 68 | 5 \| 68 | 130^A^ 420^T^ |
| ApidaeF(5-3): AGATGGGGGCATTCGTATTG |  |  |  |  |
| ApidaeR(5-3): ATCTGATCGCCTTCGAACCT |  |  |  |  |
| SEF(5-3): CTTTTGGTCGGTGGAGTGAT |  |  |  |  |
| SER(5-3): GGACGTAATCGGCACAGTTT |  |  |  |  |
| ***Neogregarines ^2^*** | 2 \| 94 | 35x 30 \| 94  30 \| 60.7 45 \| 68 | 3 \| 68 | 260 |
| NeoF (5-3): CCAGCATGGAATAACATGTAAGG |  |  |  |  |
| NeoR(5-3): GACAGCTTCCAATCTCTAGTCG |  |  |  |  |
| ***Nosema apis^A^, N ceranae^Nc 3^*** | 4 \| 95 | 35x 60 \| 95 60 \| 60 60 \| 68 | 5 \| 68 | 297^Na^ 662^Nc^ |
| NosaRNAPol-F2 (5-3): AGCAAGAGACGTTTCTGGTACCTCA |  |  |  |  |
| NosaRNAPol-R2 (5-3): CCTTCACGACCACCCATGGCA |  |  |  |  |
| NoscRNAPol-F2 (5-3): TGGGTTCCCTAAACCTGGTGGTTT |  |  |  |  |
| NoscRNAPol-R2 (5-3): TCACATGACCTGGTGCTCCTTCT |  |  |  |  |
| ***Nosema bombi ^4^*** | 4 \| 95 | 35x 60 \| 95 60 \| 50 60 \| 68 | 4 \| 68 | 323 |
| Nbombi-SSU-Jf1 (5-3): CCA TGC ATG TTT TTG AAG ATT ATT AT |  |  |  |  |
| Nbombi-SSU-Jr1 (5-3): CAT ATA TTT TTA AAA TAT GAA ACA ATA A |  |  |  |  |

**Table S4:** Summary of bee species and functional traits. Sample size refers to bees for which we had complete functional trait information, including sex (*n* = 289). Peak activity and Activity range in Julian days.

**Table S5:** Post-hoc comparison of differences between genera. Comparisons that resulted in *P* values ≥ 0.9 are not shown. The estimate correspond to the Estimated Marginal Means.

| **Pathogen** | **Contrast** | **Estimate** | **SE** | **z ratio** | ***P value*** |
| --- | --- | --- | --- | --- | --- |
| Trypanosomes | *Augochlorella - Lasioglossum* | 2.52 | 0.60 | 4.16 | 0.005 |
| Trypanosomes | *Augochlorella - Bombus* | 1.91 | 0.50 | 3.85 | 0.017 |
| Trypanosomes | *Augochlorella - Apis* | 3.17 | 0.88 | 3.61 | 0.040 |
| Trypanosomes | *Halictus - Lasioglossum* | 1.69 | 0.54 | 3.15 | 0.158 |
| Trypanosomes | *Agapostemon - Apis* | 3.34 | 1.07 | 3.12 | 0.174 |
| Trypanosomes | *Agapostemon - Lasioglossum* | 2.68 | 0.86 | 3.10 | 0.180 |
| Trypanosomes | *Lasioglossum - Melissodes* | -1.61 | 0.52 | -3.07 | 0.195 |
| Trypanosomes | *Apis - Halictus* | -2.35 | 0.83 | -2.84 | 0.328 |
| Trypanosomes | *Apis - Melissodes* | -2.27 | 0.82 | -2.76 | 0.386 |
| Trypanosomes | *Bombus - Halictus* | -1.09 | 0.41 | -2.68 | 0.443 |
| Trypanosomes | *Bombus - Melissodes* | -1.01 | 0.38 | -2.66 | 0.459 |
| Trypanosomes | *Agapostemon - Bombus* | 2.08 | 0.79 | 2.63 | 0.479 |
| Trypanosomes | *Augochlorella - Ceratina* | 1.68 | 0.67 | 2.50 | 0.585 |
| Trypanosomes | *Augochlorella - Hylaeus* | 1.66 | 0.73 | 2.27 | 0.754 |
| Trypanosomes | *Agapostemon - Ceratina* | 1.84 | 0.89 | 2.07 | 0.873 |
| *N. ceranae* | *Anthophora - Bombus* | 2.22 | 0.64 | 3.47 | 0.063 |
| *N. ceranae* | *Bombus - Melissodes* | -1.49 | 0.47 | -3.15 | 0.161 |
| *N. ceranae* | *Bombus - Megachile* | -3.84 | 1.26 | -3.06 | 0.200 |
| *N. ceranae* | *Augochlorella - Megachile* | -3.97 | 1.43 | -2.78 | 0.371 |
| *N. ceranae* | *Anthophora - Lasioglossum* | 1.91 | 0.70 | 2.75 | 0.394 |
| *N. ceranae* | *Lasioglossum - Megachile* | -3.53 | 1.29 | -2.74 | 0.397 |
| *N. ceranae* | *Bombus - Halictus* | -1.37 | 0.51 | -2.67 | 0.452 |
| *N. ceranae* | *Anthophora - Augochlorella* | 2.35 | 0.94 | 2.50 | 0.582 |
| *N. ceranae* | *Apis - Bombus* | 1.30 | 0.57 | 2.28 | 0.748 |
| *N. ceranae* | *Ceratina - Megachile* | -2.93 | 1.35 | -2.17 | 0.822 |
| *N. ceranae* | *Lasioglossum - Melissodes* | -1.18 | 0.57 | -2.08 | 0.866 |

**Table S6:** Summary table of functional traits and pathogen prevalence models. *P* values < 0.05 are in **bold**.

1 Bartomeus, I. *et al.* Historical changes in northeastern US bee pollinators related to shared ecological traits. *Proc. Natl. Acad. Sci. USA* **110**, 4656-4660, doi:10.1073/pnas.1218503110 (2013).

2 Meeus, I., De Graaf, D., Jans, K. & Smagghe, G. Multiplex PCR detection of slowly‐evolving trypanosomatids and neogregarines in bumblebees using broad‐range primers. *J. Appl. Microbiol.* **109**, 107-115 (2010).

3 Gisder, S. & Genersch, E. Molecular differentiation of *Nosema apis* and *Nosema ceranae* based on species–specific sequence differences in a protein coding gene. *J. Invertebr. Pathol.* **113**, 1-6 (2013).

4 Klee, J., Tay, W. T. & Paxton, R. J. Specific and sensitive detection of *Nosema bombi* (Microsporidia: Nosematidae) in bumble bees (*Bombus* spp.; Hymenoptera: Apidae) by PCR of partial rRNA gene sequences. *J. Invertebr. Pathol.* **91**, 98-104 (2006).
